# Supplementary material for: Resolving the full spectrum of human genome variation using Linked-Reads
Source: Genome Res. 2019 Apr;29(4):635–45. doi: 10.1101/gr.234443.118 (PMC6442396; doi:10.1101/gr.234443.118)
Supplement: Supplemental Material [file supp_gr.234443.118_Supplemental_longranger-2.2.zip › longranger-2.2.tar.bz2!/lr2.2-public/lib/stan/cmdstan/stan/doc/api/html/structstan_1_1math_1_1promote__scalar__type.html]

Stan: stan::math::promote\_scalar\_type< T, S > Struct Template Reference


|  |  |
| --- | --- |
|  | Stan  2.5.0  probability, sampling & optimization |


- Main Page
- Related Pages
- Namespaces
- Classes
- Files

- Class List
- Class Index
- Class Hierarchy
- Class Members

All Classes Namespaces Files Functions Variables Typedefs Enumerations Enumerator Friends Macros Pages

- stan
- math
- promote\_scalar\_type

Public Types |
List of all members

stan::math::promote\_scalar\_type< T, S > Struct Template Reference

Template metaprogram to calculate a type for converting a convertible type.
More...

`#include <promote_scalar_type.hpp>`

|  |  |
| --- | --- |
| Public Types | |
| typedef T | type |
|  | The promoted type. More... |
|  | |

## Detailed Description

### template<typename T, typename S> struct stan::math::promote\_scalar\_type< T, S >

Template metaprogram to calculate a type for converting a convertible type.

This is the base case.

Template Parameters
:   |  |  |
    | --- | --- |
    | T | result scalar type. |
    | S | input type |

Definition at line 19 of file promote\_scalar\_type.hpp.

## Member Typedef Documentation

template<typename T, typename S>

typedef T stan::math::promote\_scalar\_type< T, S >::type

The promoted type.

Definition at line 24 of file promote\_scalar\_type.hpp.

---

The documentation for this struct was generated from the following file:

- src/stan/math/functions/promote\_scalar\_type.hpp


---

[ Stan Home Page ]

*© 2011–2014,
Stan Development Team.*
